# Does Herd Immunity Exist in Aquatic Animals?

**DOI:** 10.3390/ijms17111898

**Published:** 2016-11-15

**Authors:** Isaac F. Standish, Travis O. Brenden, Mohamed Faisal

**Affiliations:** 1 Department of Pathobiology and Diagnostic Investigation, College of Veterinary Medicine, Michigan State University, East Lansing, MI 48824, USA; standi13@msu.edu; 2 Department of Fisheries and Wildlife, College of Agriculture and Natural Resources, Michigan State University, East Lansing, MI 48824, USA; brenden@msu.edu

**Keywords:** DNA vaccine, herd immunity, fish, muskellunge

## Abstract

Viral hemorrhagic septicemia virus genotype IVb (VHSV-IVb) is presently found throughout the Laurentian Great Lakes region of North America. We recently developed a DNA vaccine preparation containing the VHSV-IVb *glycoprotein* (*G*) gene with a cytomegalovirus (CMV) promoter that proved highly efficacious in protecting muskellunge (*Esox masquinongy*) and three salmonid species. This study was conducted to determine whether cohabitation of VHSV-IVb immunized fishes could confer protection to non-vaccinated (i.e., naïve) fishes upon challenge. The experimental layout consisted of multiple flow-through tanks where viral exposure was achieved via shedding from VHSV-IVb experimentally infected muskellunge housed in a tank supplying water to other tanks. The mean cumulative mortality of naïve muskellunge averaged across eight trials (i.e., replicates) was significantly lower when co-occurring with immunized muskellunge than when naïve muskellunge were housed alone (36.5% when co-occurring with vaccinated muskellunge versus 80.2% when housed alone), indicating a possible protective effect based on cohabitation with vaccinated individuals. Additionally, vaccinated muskellunge when co-occurring with naïve muskellunge had significantly greater anti-VHSV antibody levels compared to vaccinated muskellunge housed alone suggesting that heightened anti-VHSV antibodies are a result of cohabitation with susceptible individuals. This finding could contribute to the considerably lower viable VHSV-IVb concentrations we detected in surviving naive muskellunge when housed with vaccinated muskellunge. Our research provides initial evidence of the occurrence of herd immunity against fish pathogens.

## 1. Introduction

Hedrich [1] introduced the concept of “herd immunity” following research involving measles outbreaks in humans. As part of that research, it was determined that epidemics declined when 68% of children under 15 years of age developed immunity against measles [1]. Since this initial research, herd immunity and the associated critical vaccination threshold necessary to illicit this immunity has been investigated for both human and veterinary practices. The herd-immunity threshold is dependent on the number of secondary infections (*R*_o_), which is variable for each pathogen and environment. For example, during attempts to eradicate wild polioviruses, 100% herd immunity was accomplished with just 65% to 70% immunization coverage in North America [2], while the same vaccine regimen in South America and India resulted in only an estimated 70% herd immunity [3]. For highly pathogenic viruses, a high *R*_o_ results in a greater critical immunization threshold. In the case of measles, upwards of 92% to 95% vaccination coverage was predicted as being necessary for eradication of the disease [4]. This critical threshold has been investigated for numerous terrestrial pathogens; however, little work has been conducted on aquatic pathogens apart from simple simulation exercises [5]. Whether the concept of herd immunity is even applicable in an aquatic setting is not presently known.

Given that many vaccine preparations have been developed against aquatic pathogens, it is somewhat surprising that aquatic herd immunity has received relatively little attention. Internationally, vaccines are already in use in commercial aquaculture. Recently, Canada approved a DNA vaccine against infectious hematopoietic necrosis virus (IHNV) [6]. Similar DNA vaccines encoding the *glycoprotein* (*G*) gene of VHSV genotype I were found to be efficacious in conferring protection to salmonids following virus challenge [7,8,9]. Numerous studies have subsequently demonstrated the VHSV G protein to be the major target protein for neutralizing and protective antibodies [10,11,12,13]. Though, it is unclear whether this immune response confers any protection to neighboring individuals.

In the early 2000s, a novel genotype (IVb) of viral hemorrhagic septicemia virus (VHSV) was isolated in the Laurentian Great Lakes region of North American [14] and found to be highly pathogenic to numerous Great Lakes fish species [15,16,17,18]. Since its first detection, VHSV-IVb has spread to each of the Great Lakes and inland waterbodies throughout the region and caused multiple mass mortality events of fish populations [19]. The number of VHSV-IVb susceptible Great Lakes species is presently 28 [20], with reports documenting variability in disease course and susceptibility among species [16,17]. Recently, a VHSV-IVb vaccine preparation was developed [21] in an effort to protect aquaculture facilities against the spread of VHSV-IVb, but also with the goal of developing a vaccination program that might be used to protect wild fish populations. The vaccine consists of a DNA plasmid containing the VHSV-IVb *glycoprotein* (*G*) gene under the control of a cytomegalovirus (CMV) promoter [21,22]. The preparation has shown to be highly efficacious in protecting both muskellunge (*Esox masquinongy*) and representative Great Lakes salmonid species [22]. In muskellunge, 95% relative percent survival (RPS) was achieved following only a single administration [22]. Despite the efficacy of this VHSV-IVb vaccine, the lack of understanding as to whether a herd immunity response can be elicited in aquatic population leads to some uncertainty as to the potential usefulness of this vaccine in combatting outbreaks of the disease in wild populations. A successful demonstration of the concept of aquatic herd immunity would present the possibility of using the extensive hatchery system within the Great Lakes to actively combat pathogens. Hatchery propagated individuals could be immunized prior to stocking in public waters to supplement the herd immunity and establish a critical immunization threshold. To this end, the goal of this study was to examine whether aquatic herd immunity against VHSV-IVb could be demonstrated in a laboratory setting.

## 2. Results

In nearly all trials (i.e., replicates), positive shedding by infected muskellunge was detected within the first week of initiation, and then in most cases decreased to near zero or below detectable limits by weeks 3 and 4 post infection, at which point all infected muskellunge had succumbed to infection. The only exceptions to this observed shedding dynamic was Trial 6 where no shedding was detected during any of the sampling events and the third trial where shedding increased from Week 1 to Week 2. During the first two weeks of each trial, infected muskellunge in the source tanks clearly showed signs of acute VHSV-IVb infection, including extensive petechial hemorrhage along dorsal surface and mortalities exhibited extensive hemorrhage throughout the musculature, liver, swim bladder and renal mesentery.

Within two weeks following the initiation of each trial, signs of VHSV-IVb infection were observed in muskellunge held in the downstream tanks, particularly in muskellunge from the all naïve treatments. Numerous fish exhibited severe petechial hemorrhage, erratic swimming, and pale gills. In most trials, the observation of these morbid individuals was followed by a steep increase in mortalities. In all trials, VHSV-IVb was re-isolated from all mortalities.

We found no significant difference in shedding by infected muskellunge among the trials (*F* = 0.23; df = 7, 14; *p*-value = 0.9706). Despite this lack of a significant difference, we still chose to include log_e_ + 1 transformation of shedding of infected muskellunge in the trials as a covariate in the mixed-effect models.

### 2.1. Cumulative Mortality

The highest mortalities in the experiment involved naïve muskellunge housed alone, ranging from 30% to 100%, with an average cumulative mortality of 80.2% (Table 1). Conversely, vaccinated muskellunge stocked alone experienced the lowest mortality rates during the experiment, with mortality rates in all cases being less than 16.7% and an average cumulative mortality of 3.1% (RPS = 96.2%) (Table 1) The cumulative mortality of naïve muskellunge stocked with vaccinated muskellunge ranged from 0% to 100% with an overall average of 36.0% (RPS = 55.1%) (Table 1). The cumulative mortality of vaccinated muskellunge housed with naïve muskellunge ranged from 0.0% to 50.0% with an overall average of 6.0% (RPS = 92.5%) (Table 1).

There was an overall significant difference among the housing combination × organism of interest interaction (*F* = 12.81; df = 3, 21.39; *p*-value < 0.0001). The estimated coefficient for shedding of infected muskellunge was 0.0619 (SE = 0.1786), which was not significantly different from zero (*F* = 0.12, df = 1, 7.02; *p*-value = 0.7388). The estimated variance for the trial effect was 1.539 (SE = 1.552), whereas the variance for the trial × housing combination × organism of interest interaction was 2.009 (SE = 1.216).

Cumulative mortality was significantly greater in naïve muskellunge compared to vaccinated muskellunge when housed separately (Table 2). Based on the predicted marginal means of cumulative mortality from the fitted model, the odds of naïve muskellunge housed alone succumbing during the experiment was approximately 455 times greater than that of vaccinated muskellunge housed alone (Table 2). Cumulative mortality was also significantly greater in naïve muskellunge housed alone compared to naïve muskellunge housed with vaccinated muskellunge (Table 2). The odds of naïve muskellunge housed alone succumbing was approximately 14 times greater than that of naïve muskellunge housed with vaccinated muskellunge based on the fitted model (Table 2). We did not find a significant difference in cumulative mortality between vaccinated muskellunge housed alone and vaccinated muskellunge co-occurring with naïve muskellunge (Table 2).

### 2.2. Circulating Anti-VHSV Antibodies

The highest OD values were observed in vaccinated muskellunge when housed with naïve muskellunge (mean = 1.04) (Table 3), whereas the lowest OD values were observed in naïve muskellunge when housed with vaccinated muskellunge (mean = 0.11). Mean OD values for the other housing combinations ranged from 0.21 for naïve muskellunge stocked along to 0.28 for vaccinated muskellunge stocked alone.

There was an overall significant difference in OD values among the housing combination × organism of interest interaction levels (*F* = 20.64; df = 3, 6.84; *p*-value = 0.0008). The estimated coefficient for shedding of infected muskellunge was 0.103 (SE = 0.051), which was not significantly different from zero (*F* = 4.07, df = 1, 2.334; *p*-value = 0.1627). The estimated variance for the trial effect was 0.111 (SE = 0.171), whereas the variance for the trial × stocking combination × organism of interest interaction was 0.011 (SE = 0.113). Pairwise comparisons indicated that the OD values for vaccinated muskellunge housed with naïve muskellunge were significantly greater than those from all other housing combination × organism of interest levels (Table 4). We did not find significant differences in OD values for any of the other pairwise comparisons (Table 4).

### 2.3. Viable VHSV-IVb Concentrations in Survivors

Viable VHSV-IVb concentrations in surviving fish were the greatest in naïve muskellunge housed alone. The average concentration was 16.8 pfu·mg^−1^, with VHSV-IVb detected in 10 of 21 surviving individuals (Table 5). Conversely, 12 out of 20 of the surviving naïve muskellunge when housed with vaccinated muskellunge were found to be actively infected with VHSV-IVb, although, in this case, the average concentration was 3.0 pfu·mg^−1^. Viable VHSV-IVb concentrations were detected in 10 of 19 of the surviving vaccinated muskellunge housed with naïve muskellunge with an overall average concentration of 0.7 pfu·mg^−1^. Meanwhile, viral concentrations were lowest in vaccinated muskellunge housed alone, detecting viable virus in only 3 of 35 individuals and a mean concentration of 0.2 pfu·mg^−1^.

There was a significant difference in the percent positive VHSV-IVb detections among the housing combination × organism of interest interaction levels (*F* = 5.09; df = 3, 7.261; *p*-value = 0.0335). The estimated coefficient for shedding of infected muskellunge was 0.088 (SE = 0.100), which was not significantly different from zero (*F* = 0.77, df = 1, 3.812; *p*-value = 0.4307). The estimated variance for the trial effect was 0.271 (SE = 0.534), whereas the variance for the trial × stocking combination × organism of interest inte raction was 0.061 (SE = 0.488). Pairwise comparisons indicated that the percent positive VHSV-IVb detections for vaccinated muskellunge was significantly lower than all other housing combination × organism of interest levels (Table 6). We did not find significant differences in percent positive detections for any of the other pairwise comparisons (Table 6).

We observed no significant difference in VHSV-IVb titers among the housing combination × organism of interest interaction levels (*F* = 2.18; df = 3, 8.418; *p*-value = 0.1641). We attribute the lack of an overall difference in viral concentrations in survivors from the housing combinations to the high number of zero concentrations that were observed. The estimated coefficient for shedding of infected muskellunge was 0.0259 (SE = 0.08802), which was not significantly different from zero (*F* = 0.09, df = 1, 2.298; *p*-value = 0.7932). The estimated variance for the trial effect was 0.387 (SE = 0.494), whereas the variance for the trial × housing combination × organism of interest interaction was 0.333 (SE = 0.206).

## 3. Discussion

This study was designed to examine whether aquatic herd immunity against VHSV-IVb could be elicited in a laboratory setting. The initial results demonstrate that when naïve muskellunge co-occur with vaccinated muskellunge at an equal abundance level, naïve muskellunge experience significant protection and a 55.1% RPS rate. Thus, these results suggest that herd immunity can be elicited in an aquatic setting. The vaccinated muskellunge housed with naïve muskellunge exhibited a vigorous humoral response with significantly higher OD values than those obtained from vaccinated muskellunge alone. This heightened immune response likely resulted from increased viral exposure from co-mingling with the naïve muskellunge following challenge.

Tanks containing vaccinated and naïve muskellunge housed together or alone were exposed to VHSV-IVb via shedding from infected muskellunge in a common-source tank, which mimics a natural course of exposure. Additionally, this method of exposure allowed viral concentrations to naturally vary and the controlled distribution of water allowed differing tanks to be exposed to identical viral concentrations within a trial. However, the stochasticity of viral shedding between trials also accounts for the variability in mortality and RPS of naïve muskellunge housed with vaccinated muskellunge. The small number of fish per tank likely also attributed to the variability in mortality, though utilizing numerous study replicates indicates a protective effect from comingled. Subsequent studies will undoubtedly need to increase the fish sample sizes and examine additional species to support these findings.

While the herd immunity concept seems to exist in the aquatic environment, how it results may require additional investigations as they were beyond the scope of this study. For example, pioneering work demonstrated that teleost mucosal surfaces harbor both IgM and IgT immunoglobulis [23,24], both of which can neutralize the virus in the skin and gill mucus layer of vaccinated fish. It is also possible, though not scientifically proven, that both antibody types can be shed into the water column resulting in viral neutralization by the vaccinated individuals. Regardless, if the immunoglobulins are bound to mucus or shed in the water, they can account for decreased viral transmission. In this fashion, simply stocking immunized individuals into an aquatic system would aid to the establishment of the critical immune threshold that is beneficial to both vaccinated (as it increases their antibody responses) and naïve (as it improves survival) fish.

Few researchers have examined the concept of herd immunity in aquatic environments as epidemics can be difficult to visualize and quantify. Epidemics occur when a high number of susceptible individuals leads to efficient transmission or contact (i.e., a high *R*_o_ value). The *R*_o_ value is linked to a critical immunity threshold which is the proportion of the population that must be immune or vaccinated in order to prevent further transmission of a pathogen [25]. The *R*_o_ and transmission dynamics are largely uninvestigated for aquatic pathogens such as VHSV. Moreover, disease dynamics vary based on virulence, host susceptibility and behavior, innate immunity, contact rates, environmental conditions, etc. In addition, for VHSV, shedding rates can be transient and differ on the order of magnitudes [26]. This variability complicates the calculation of *R*_o_, however, the results of this study can be used to forgo this calculation and inform a modeling effort to assess aquatic herd immunity and the number of fish needed to be stocked to elicit a herd immunity effect under a range of conditions on a much larger scale than possible in a laboratory setting.

## 4. Materials and Methods

### 4.1. Experimental Fish and Care

All fish used in the included study were certified disease free in accordance to World Organization for Animal Health (OIE) testing guidelines [27] prior to use. Two groups of juvenile muskellunge were used throughout the study. The first group was used for the first four experimental trials and was obtained 14 weeks post-hatch (average 14.2 cm (SD = 1.4), 11.9 g (SD = 3.8)) from the Chautauqua State Fish Hatchery (New York Department of Environmental Conservation, Chautauqua, NY, USA). The second group of juvenile muskellunge was used in the remaining experimental trials and was obtained 16 weeks post-hatch (average 12.7 cm (SD = 0.9), 16.1 g (SD = 3.7)) from the Wolf Lake Fish Hatchery (Michigan Department of Natural Resources, Mattawan, MI, USA). All muskellunge were fed live fathead minnows (*Pimephales promelas*) obtained from Anderson Farms Inc. (Lonoke, AR, USA) and certified as free of important disease. An additional 60 minnows were necropsied and underwent additional testing according to the American Fisheries Society Fish Health Section [28].

All experimental fishes were acclimated in a 500-L circular fiberglass tank in a continuous flow-through system with facility-chilled well water and supplemental aeration. Fish were housed and all experiments were conducted in the University Containment facility (Michigan State University, East Lansing, MI, USA). Fish were fed ad libitum throughout study except for the 1st week post-viral challenge when food was withheld. Two weeks prior to immunization, randomly selected fish were transferred and acclimated to 72-L polyethylene flow-through tanks (Pentair Aquatic Eco-Systems, Apopka, FL, USA) containing supplemental aeration.

The care and use of laboratory animals were followed in accordance with the ethical guidelines defined by Michigan State University’s (MSU) Institutional Animal Care and Use Committee (AUF 03/14-047-00).

### 4.2. Virus and Cell Culture

The VHSV-IVb isolate used throughout this study was the Great Lakes index strain MI03 [14]. The isolate has been maintained by continuous subculture in the cell line Epithelioma papulosum cyprini (EPC). The EPC cell line was maintained and subcultured in 150 cm^2^ tissue culture flasks (Corning) at 25 °C using a basal media of Earle’s salt-based minimal essential medium (MEM) (Invitrogen, Carlsbad, CA, USA) and supplemented with 29.2 mg·mL^−1^ l-glutamine (Invitrogen), penicillin (100 IU·mL^−1^) (Invitrogen), streptomycin (0.1 mg·mL^−1^) (Invitrogen), 10% fetal bovine serum (Gemini BioProducts, West Sacramento, CA, USA), and sodium bicarbonate (7.5% *w*/*v*) (Sigma, St. Louis, MO, USA). Viable viral concentrations were determined using plaque assay on EPC cell line using polyethylene glycol and a methylcellulose overlay [29,30]. Virus was then aliquoted into cryogenic vials (Corning Inc., Tewksbury, MA, USA) for one time use and stored at −80 °C until needed.

### 4.3. Construction of pVHSivb-G Plasmid

The pcDNA 3.1 (+) is a commercially available vector containing the human CMV immediate-early promoter. The DNA vaccine construct containing the VHSV-IVb *G* gene, designated pVHSivb-G, [21,22], was modeled after successful DNA vaccines against VHSV genotype I [7,31] and IHNV [32]. The construction and production of this plasmid were outsourced to Life Technologies (Carlsbad, CA, USA). In brief, an *Eco*RI restriction site (G/AATTC) followed by a kozak consensus sequences terminating with the first amino acid of the complete MI03GL VHSV-IVb isolate *G* gene (1524 bp) was synthesized. An *Xba*I restriction site (T/CTAGA) was then added following the 3’ termination codon. The assembled fragment was then digested using the described endonucleases and sub-cloned into the eukaryotic expression vector pcDNA 3.1(+) (Invitrogen). The plasmid was transformed and propagated into K12 *Escherichia coli*. Sequencing confirmed the correct *glycoprotein* gene sequence and orientation. The final vector, designated pVHSivb-G, was diluted to 1 mg·mL^−1^ in sterile phosphate buffered saline (PBS) and stored at −80 °C.

### 4.4. Experimental Design

This experiment involved eight replicate trials housing naïve and vaccinated muskellunge separately or in combination and exposing them to VHSV-IVb via virus shedding from infected muskellunge. It was anticipated that there could be considerable variation in the measured responses of this experiment because of our trying to mimic a natural course of exposure to VHSV (see below); the high number of replicate trials was therefore needed to separate treatment effects from the variability across trials [33]. Vaccinated muskellunge were inoculated according to a pre-developed regime that in previous studies resulted in an average RPS of 95% [22]. Immediately prior to vaccination, vectors were thawed and diluted to 10 μg in 100 μL of sterile PBS. Individual fish were randomly allocated to treatment tanks prior to anesthetization with 0.1 g·L^−1^ of tricaine methanesulfonate (MS-222) (Western Chemical, Ferndale, WA, USA), buffered with 0.3 g·L^−1^ sodium bicarbonate. Muskellunge were vaccinated intramuscularly with 10 μg of the pVHSivb-G plasmid in the left epaxial muscle slightly posterior to the pectoral fins. Vaccinated individuals that would be housed in tanks with naïve individuals were intramuscularly marked with 9-mm passive integrated transponder (PIT) tags (Biomark^©^ Inc., Boise, ID, USA) so that naïve and vaccinated could be identified. A pool of muskellunge was vaccinated at six-week intervals and maintained 72-L polyethylene flow-through tanks (Pentair Aquatic Eco-Systems, Apopka, FL, USA). Following vaccination, fish were allowed to react to the antigen for 1880 degree days (20 weeks at 13 °C).

The experimental layout for this research consisted of three 72-L polyethylene flow-through tanks containing supplemental aeration that all received water flow from a single common source tank. Water initially flowed into a tank containing infected muskellunge. Naïve muskellunge obtained in the original batch of muskellunge from Chautauqua State Fish Hatchery were used as shedders throughout all trials. Muskellunge were intraperitoneally (IP) infected with a low dose of VHSV-IVb that previous studies indicated would elicit shedding [17,26]. To infect muskellunge, immediately prior to infection, muskellunge were anesthetized as previously described. Thawed VHSV-IVb was diluted to a concentration of 1.98 pfu in 100 μL of sterile PBS and administered using intraperitoneal injection (IP). Following infection, muskellunge were held in a 72-L polyethylene flow-through tank for up to seven days until each trial was initiated. In each replicate trial the number of initial infected muskellunge placed into the shedder tank was equal to the number of downstream tanks i.e., three infected muskellunge for three downstream tanks. Once an infected individual succumbed, that individual was removed and not replaced. Additionally, if all the infected individuals succumbed, water continued to flow through the empty shedder tank.

The water from the tank containing infected individuals was then equally distributed to downstream tanks. A total of 8 replicate trials were conducted. All trials included tanks consisting solely of naïve muskellunge and vaccinated muskellunge and a tank where naïve and vaccinated muskellunge were housed together. Trials differed only slightly in the initial stocking densities, tanks contained between 10 and 14 fish·tank^−1^ so as to limit density effects on survival. In co-occurring tanks, an equal number of vaccinated and naïve muskellunge were used. The assignment of housing combinations (i.e., experimental treatments) to the tanks (i.e., experimental units) in each trial was randomly determined. Throughout all trials, tanks containing populations of naïve and vaccinated muskellunge housed together, the populations were assessed separately.

One week prior to the initiation of each trial, fish from each treatment were randomly allocated into their population tanks and acclimated prior to the introduction of virus into the system. Water temperatures were maintained at 11 ± 1 °C throughout all trials through the use of a chiller system. Following the introduction of virus into the system, the experiment was run for 28 days during which each population was monitored for morbidity and mortality. The only exception to this was Trial 4, which was run for 60 days due to low mortality rates in any of the housing combinations. Moribund fish were allowed to actively shed and infect other individuals rather than being removed from their respective tanks.

### 4.5. Response Measures

#### 4.5.1. Mortalities

All mortalities were necropsied and kidney, spleen and heart samples were aseptically collected and homogenized using a Biomaster Stomacher (Wolf Laboratories Ltd., Pocklington, UK) on high speed for 2 min. Homogenates were diluted 1:4 (*w*/*v*) with MEM, supplemented with 12 mM tris buffer (Sigma), penicillin (100 IU·mL^−1^), streptomycin (100 µg·mL^−1^) and Amphotericin B (250 µg·mL^−1^) (Invitrogen). Samples were centrifuged at 2700× *g* for 30 min at 4 °C and inoculated onto EPC monolayers. After 14 days supernatant was removed, frozen at −80 °C, thawed at centrifuged at 2700× *g* for 15 min at 4 °C. Supernatant was then re-infected onto fresh EPC monolayer and incubated for 14 days before being examined for viral cytopathic effect (CPE). The presence of VHSV-IVb was confirmed using real-time reverse transcription polymerase chain reaction (RT-PCR) assay specific for VHSV-IVb [34,35]. The cumulative mortality of the tank containing all naïve muskellunge in each trial was used to calculate RPS of vaccinated or naïve muskellunge housed with vaccinated [36].

$$\mathrm{RPS}=1.0-\left(\frac{\%\;\mathrm{cumulative}\;\mathrm{mortality}\;\mathrm{vaccinated}}{\%\;\mathrm{cumulative}\;\mathrm{mortality}\;\mathrm{of}\;\mathrm{naive}}\right)\times \;100\%$$

#### 4.5.2. VHSV-Shedding

Viral shedding rates of the IP infected muskellunge were assessed once a week during each trial for as long as individuals remained. Shedding rates were used as a covariate for explaining variability in results among the trials. Shedding was assessed using a modified protocol of that described by Kim and Faisal [26]. First, the entire flow though system was turned off and fish remained in their respective tanks with supplemental aeration. After 90 min, water was mixed and a 50 mL water sample was taken from each tank and the flow was resumed. Water samples were stored at 4 °C until processing within 24 h. For processing, samples were vortexed and centrifuged at 2700× *g*, at 4 °C for 10 min. After centrifugation, a viral plaque assay (VPA) was conducted as previously described. After 6 days, cell monolayers were stained with crystal violet (Sigma) and 18% formaldehyde (Avantor Performance Materials Inc., Center Valley, PA, USA). Viral plaques were counted and the theoretical shedding rate (pfu·hour^−1^) for the tank was determined.

#### 4.5.3. Circulating Anti-VHSV Antibodies

Levels of circulating anti-VHSV antibodies in surviving muskellunge from Trials 4, 5, 6 and 8 were assessed using a newly develop indirect enzyme-linked immunosorbent assay (ELISA) [22]. At the termination of each trial, surviving muskellunge were euthanized with 0.3 g·L^−1^ of tricaine methanesulfonate buffered with sodium bicarbonate. Blood was collected using a caudal venipuncture, stored at 4 °C for 2 h and centrifuged at 2700× *g* for 10 min at 4 °C. The serum was then aliquoted and stored at −80 °C until analysis.

The indirect ELISA was conducted as previously described [22]. Briefly, sera was first heat inactivated at 45 °C for 30 min. Serum was centrifuged for 10 min at 2700× *g*, 4 °C, immediately prior to diluting in a solution of 1% nonfat dried milk in PBS (dilution of PBS-5% NFDM, Sigma). Solid phase ELISA took place in polystyrene microplates (96-well, Microlon^®^600 with chimney wells; Greiner Bio-One, Monroe, NC, USA). Plates were sealed during all incubation periods (SealPlate^®^; Sigma) and washed 5 times following each incubation unless otherwise stated using PBS containing 0.05% Tween 20 (PBS-T20; Sigma) in an automated microplate washer (BioTek, 4Lx405™ plate washer; Winooski, VT, USA).

Microtiter assay plates were coated with 100 µL·well^−1^ of purified VHSV-IVb at 1 µg·mL^−1^ and incubated overnight (14–16 h) at 4 °C in a humid chamber. After the overnight incubation, plates were washed and unbound sites were blocked with the addition for 430 µL·well^−1^ of PBS containing 5% NFDM (PBS-5%; Sigma) and incubation at 37 °C for 1 h. Heat inactivated and diluted test and control muskellunge sera was then added to duplicate wells at 100 µL·well^−1^. After incubating at 25 °C for 1 h, plates were washed and 100 µL·well^−1^ of 1:30,000 dilution of a mouse anti-muskellunge mAb (designated 3B10) was added and incubated at 25 °C for 1 h. Plates were washed and 100 µL of 1:4000 dilution of a commercially available goat anti-mouse secondary antibody horseradish peroxidase (HRP) conjugate (Invitrogen) was added to each well and incubated at 25 °C for 1 h. Plates were developed by the addition of 100 μL of 0.4 mg·mL^−1^ *o*-phenylenediamine (Sigma) in phosphate citrate buffer (Sigma) containing 3 mM hydrogen peroxide (Avantor Performance Materials Inc.). The reaction proceeded for 30 min at 25 °C in the dark. Without washing, the reaction was stopped with the addition of 50 mL of 3 M sulfuric acid (H_2_SO_4_; Avantor Performance Materials Inc.). The optical density (OD) was read at 490 nm on a BioTek, ELx808™ plate reader (BioTek) using Gen5 software (BioTek). The average value of blank wells was subtracted from test and control wells prior to analysis.

#### 4.5.4. Viable VHSV-IVb Concentrations in Survivors from Each Population

Viable viral concentrations were assessed in the survivors of Trials 4, 5, 6 and 8. At the termination of each trial, all survivors were euthanized as previously described. A sample of the posterior kidneys was collected aseptically from each individual and stored at 4 °C until processed individually within 24 h. The tissue was homogenized and diluted 1:10 (*w*/*v*) with MEM as previously described. Samples were vortexed and centrifuged at 2700× *g* for 30 min at 4 °C and supernatant was used to conduct a VPA as previously described. The number of viral plaques was used to determine viable viral concentrations (pfu·mg^−1^).

### 4.6. Data Analysis

Differences in viral shedding rates of the IP infected muskellunge among the trials were tested using one-way analysis of variance following log*_e_* + 1 transformation of the shedding rates. Each of the response measures from the experimental fish described in Section 4.5 was analyzed using generalized linear mixed-effect models. For cumulative mortality and percent positive VHSV-IVb detections in surviving fish from the trials, a binomial distribution was assumed, whereas for the other response measures a Normal distribution was assumed following either log*_e_* (circulating antibodies) or log*_e_* + 1 (viral concentration in survivors) transformation. Each of the mixed-effect models included as fixed-effect variables the housing combination × organism of interest (vaccinated or naïve) interaction and log*_e_* + 1 transformation of viral shedding of infected muskellunge in their respective trials as fixed-effect variables. The mixed effect models also included trial and trial × housing combination × organism of interest interaction as random effects. If an overall significant difference among the housing combination × organism of interest interaction was detected for a response measure, then pre-planned comparisons of particular levels of interest were conducted (e.g., survival of naïve muskellunge when housed alone versus when housed with vaccinated muskellunge). Denominator degrees of freedom for the tests of overall differences among treatments and the pre-planned pairwise comparisons were set using the Satterthwaite approximation. All analyses were conducted using PROC GLIMMIX in SAS [37].

## 5. Conclusions

In this study, we examine the concept of “herd immunity” in an aquatic system. The cumulative results from eight trails indicates that cohabitation of immunized muskellunge with naïve individuals indeed provides a protective effect to the naïve cohort. This represents an innovative concept that certainly warrants further investigation, such as the mechanism of this protective effect, and whether protection is observed with other species and pathogens. Undoubtedly, this research and subsequent examinations will provide valuable insight in disease prevention of aquatic species.

## Figures and Tables

**Table 1:** Cumulative mortality and relative percent survival (RPS). The number of fish stocked, cumulative mortality and RPS by housing combination for each of the experimental trials conducted for this research. In tanks containing naïve and vaccinated muskellunge together, the mortality and RPS are assessed separately. NA: not applicable.

Housing Combination	Number of Fish	Cumulative Mortality	RPS
Trial 1			
Naïve muskellunge	12	91.0%	NA
Vaccinated muskellunge	12	16.7%	81.9%
Naïve muskellunge with vaccinated muskellunge	6	100.0%	−9.2%
Vaccinated muskellunge with naïve muskellunge	6	50.0%	45.5%
Trial 2			
Naïve muskellunge	12	100.0%	NA
Vaccinated muskellunge	12	0.0%	100.0%
Naïve muskellunge with vaccinated muskellunge	6	83.3%	16.6%
Vaccinated muskellunge with naïve muskellunge	6	0.0%	100.0%
Trial 3			
Naïve muskellunge	12	100.0%	NA
Vaccinated muskellunge	12	0.0%	100.0%
Naïve muskellunge with vaccinated muskellunge	7	14.3%	85.7%
Vaccinated muskellunge with naïve muskellunge	7	0.0%	100.0%
Trial 4			
Naïve muskellunge	14	35.7%	NA
Vaccinated muskellunge	14	0.0%	100.0%
Naïve muskellunge with vaccinated muskellunge	7	28.6%	20.0%
Vaccinated muskellunge with naïve muskellunge	7	0.0%	100.0%
Trial 5			
Naïve muskellunge	12	83.3%	NA
Vaccinated muskellunge	12	8.3%	90.0%
Naïve muskellunge with vaccinated muskellunge	6	0.0%	100.0%
Vaccinated muskellunge with naïve muskellunge	6	0.0%	100.0%
Trial 6			
Naïve muskellunge	12	100.0%	NA
Vaccinated muskellunge	12	0.0%	100.0%
Naïve muskellunge with vaccinated muskellunge	6	16.7%	83.3%
Vaccinated muskellunge with naïve muskellunge	6	0.0%	100.0%
Trial 7			
Naïve muskellunge	12	100.0%	NA
Vaccinated muskellunge	12	0.0%	100.0%
Naïve muskellunge with vaccinated muskellunge	6	16.7%	83.3%
Vaccinated muskellunge with naïve muskellunge	6	0.0%	100.0%
Trial 8			
Naïve muskellunge	10	30.0%	NA
Vaccinated muskellunge	12	0.0%	100.0%
Naïve muskellunge with vaccinated muskellunge	6	33.3%	−11.1%
Vaccinated muskellunge with naïve muskellunge	6	0.0%	100.0%

**Table 2:** Pairwise comparison of cumulative mortality. Results from pairwise comparisons of cumulative mortality between tank treatments. The *t*-statistic, degrees of freedom, and *p*-value are included for each comparison. The odds ratio (OR) and upper and lower 95% confidence limit for the ORs are also shown. The ORs measure how much more likely fish in housing combination 1 would experience mortality versus individuals in housing combination 2. ORs were calculated using the predicted marginal mean cumulative mortalities from the fitted model.

Housing Combination 1	Housing Combination 2	*t*-Statistic	df	*p*-Value	Odds Ratio	OR 95% LCL	OR 95% UCL
Naïve muskellunge	Vaccinated muskellunge	5.41	25.47	<0.0001	455.43	44.42	>999.99
Naïve muskellunge	Naïve muskellunge with vaccinated muskellunge	2.95	13.11	0.0112	14.01	2.03	96.6
Vaccinated muskellunge	Vaccinated muskellunge with naïve muskellunge	−0.25	27	0.8008	0.70	0.04	11.93

**Table 3:** Anti-VHSV-IVb antibodies following exposure. OD values indicating the presence of anti-VHSV-IVb antibodies in challenged muskellunge sera collected from muskellunge kept at different housing combinations at the termination of cohabitation Trials 4, 5, 6 and 8. The mean OD values were determined using an indirect enzyme-linked immunosorbent assay (ELISA) and were calculated by averaging over survivors within individual tanks and then averaging across tanks.

Post-Exposure Survivors	Samples	Mean OD Value
Naïve muskellunge	18	0.21 (SD = 0.16)
Vaccinated muskellunge	38	0.28 (SD = 0.17)
Naïve muskellunge with vaccinated muskellunge	19	0.11 (SD = 0.08)
Vaccinated muskellunge with naïve muskellunge	25	1.04 (SD = 0.64)

**Table 4:** Pairwise comparison of anti-VHSV-IVb antibody levels. Pairwise comparisons of circulating binding anti-VHSV-IVb antibody OD values in surviving muskellunge following VHSV-IVb exposure. OD values were modeled using a mixed effect model following log_e_ transformation of the data. The *t*-statistic, degrees of freedom, and *p*-value are shown for each comparison.

Housing Combination 1	Housing Combination 2	*t*-Statistic	df	*p*-Value
Naïve muskellunge	Naïve muskellunge with vaccinated muskellunge	0.89	8.91	0.3949
Naïve muskellunge	Vaccinated muskellunge	−1.25	5.33	0.2626
Naïve muskellunge	Vaccinated muskellunge with naïve muskellunge	−5.79	6.97	0.0007
Naïve muskellunge with vaccinated muskellunge	Vaccinated muskellunge with naïve muskellunge	−7.07	10.53	<0.0001
Vaccinated muskellunge	Vaccinated muskellunge with naïve muskellunge	−5.67	5.67	0.0016

**Table 5:** VHSV-IVb concentrations in survivor tissues. Number of positive detections and mean viable VHSV-IVb concentrations in the posterior kidney of surviving muskellunge from cohabitation Trials 4, 5, 6 and 8. Plaques were enumerated using a VPA as previously described. The mean viral concentrations were calculated by averaging over survivors within individual tanks and then averaging across tanks.

Housing Combination	Samples	Positive	Mean Viral Concentration (pfu·mg^−1^)
Naïve muskellunge	21	10	16.8 (SD = 27.0)
Vaccinated muskellunge	35	3	0.2 (SD = 0.4)
Naïve muskellunge with vaccinated muskellunge	20	12	3.0 (SD = 5.3)
Vaccinated muskellunge with naïve muskellunge	19	10	0.7 (SD = 0.3)

**Table 6:** Pairwise comparison of VHSV-IVb concentrations in survivors. Pairwise comparisons of percent positive VHSV-IVb detections in surviving muskellunge following viral exposure. The *t*-statistic, degrees of freedom, and *p*-value are shown for each comparison.

Housing Combination 1	Housing Combination 2	*t*-Statistic	df	*p*-Value
Naïve muskellunge	Naïve muskellunge with vaccinated muskellunge	−0.46	5.47	0.6637
Naïve muskellunge	Vaccinated muskellunge	3.11	8.16	0.0141
Naïve muskellunge	Vaccinated muskellunge with naïve muskellunge	−0.01	4.80	0.9900
Naïve muskellunge with vaccinated muskellunge	Vaccinated muskellunge with naïve muskellunge	0.48	7.30	0.6461
Vaccinated muskellunge	Vaccinated muskellunge with naïve muskellunge	−3.21	11.71	0.0077
